# Research on the relationship between executive cognition and innovation performance of SRDI SMEs

**DOI:** 10.3389/fpsyg.2025.1493242

**Published:** 2025-03-28

**Authors:** Yan Chen, Huilin Du, Dehan Wang

**Affiliations:** ^1^Business School, Shandong University of Technology, Zibo, China; ^2^College of Economics and Management, East China Normal University, Shanghai, China; ^3^Yantai Research Institute, China Agricultural University, Yantai, Shandong, China

**Keywords:** executive cognition, compositional capability, innovation performance, organization routine updating, SRDI SMEs

## Abstract

**Purpose:**

This study aimed to explore the relationship between executive cognition and the innovation performance of specialized, refined, distinctive, and innovative small and medium sized enterprises (SRDI SMEs) in China, establish a theoretical framework of the influence mechanism of executive cognition on innovation performance of SRDI SMEs, and give some management implications for SRDI SMEs to improve innovation performance.

**Design, methodology, and approach:**

Through theoretical derivation, 409 valid questionnaires from 98 SRDI SMEs in China and statistical analysis techniques, this paper studied the effect of executive cognition of SRDI SMEs on innovation performance and its mechanism.

**Findings:**

This study found that executive cognition has a significant positive impact on innovation performance, compositional capability plays an intermediary role between executive cognition and innovation performance, and organization routine updating enhances the positive relationship between executive cognition and compositional capability.

**Originality and value:**

This study was among the first to investigate the relationship between executive cognition and innovation performance of SRDI SMEs.

## 1 Introduction

Specialized, Refined, Distinctive, and Innovative Small and Medium-sized Enterprises (SRDI SMEs) represent the most dynamic segment within the SME sector. These enterprises are the core driving force behind technological breakthroughs, green transformations, global supply chain stabilization, and the low-carbon transition of the global economy (Lian, 2024). However, SRDI SMEs currently face significant developmental barriers, including capital constraints, financing difficulties, supply chain disruptions, and heightened uncertainties in the business environment (Lian, 2024). Innovation is the essence of SRDI SMEs and a vital pathway for overcoming these challenges and achieving sustainable growth (Yang and Xu, 2024). By leveraging innovation, SRDI SMEs can achieve technological differentiation and establish competitive advantages in niche markets. Concurrently, innovation can facilitate intellectual property financing, accelerate digital transformation, and enhance global strategic deployment, thereby strengthening the core competitiveness of these enterprises (Yang and Xu, 2024). Therefore, effectively enhancing innovation performance in a dynamic environment is an urgent issue that SRDI SMEs must address.

Senior leaders are key decision-makers and drivers of organizational innovation initiatives. Their cognitive abilities inevitably exert a significant influence on overall corporate innovation performance (Malhotra and Harrison, 2022; Zhou and Ding, 2024;). Existing studies grounded in the Upper Echelons Theory have explored the relationship between executive cognition and innovation performance, demonstrating that enhancing executive cognition can significantly improve corporate innovation performance (Malhotra and Harrison, 2022; Zhou and Ding, 2024). For instance, Bajaba et al. (2022) proposed that executive cognition can significantly enhance innovation quality. Maitlis and Sonenshein (2010) demonstrated that during financial crises, Top Management Teams (TMTs) could drive strategic innovation by adjusting cognitive frameworks through sense-making processes. Empirical evidence also shows that executive cognition significantly enhances technological innovation in high-tech industries (McEvily and Zaheer, 1999; Barker and Mueller, 2002; Arena et al., 2018). Additionally, Wang et al. (2022) validated that internationalized cognition among executives drives exploratory innovation. However, the connection between executive cognition and innovation performance, as well as the underlying mechanisms in SRDI SMEs, has not received adequate attention. According to the Upper Echelons Theory, executive cognition plays a critical role in decision-making, strategic selection, and implementation in the corporate innovation process (Hambrick and Mason, 1984; Adner and Helfat, 2003). Particularly for SRDI SMEs, only high executive cognition can support them in making correct decisions in a harsh business environment (Shi and Wu, 2022). The essence of innovative activities can be conceived as an ongoing process of creating novel outcomes through the integration of resources (Drucker, 2002), where the resource integration approach hinges upon the decisions of senior leaders. Thus, this study posits a positive relationship between executive cognition and innovation performance in SRDI SMEs.

SRDI SMEs often adopt a Focus Strategy to pursue professional development due to their relative shortage of resources (Chen et al., 2023). Due to their deep cultivation of market segments, SRDI SMEs are often constrained by “bottleneck” issues in the process of sustainable development (Shi and Wu, 2022). At the same time, their small scale makes it more challenging for them to obtain heterogeneous resources, necessitating the need to maximize resource utility through effective integration of common resources (Shan et al., 2021). Lu and Sun (2013), influenced by Chinese philosophical concepts such as “harmony,” “symbiosis,” and “moderation,” proposed the Composition-based View, arguing that for enterprises lacking heterogeneous resources, innovation performance can be improved through the creative compound utilization of existing internal and external resources. They then introduced the concept of compositional capability, defined as the unique ability of an enterprise to synergistically integrate existing tangible or intangible resources from internal and external sources. Additionally, the Upper Echelons Theory suggests that higher levels of executive cognition can help enterprises enhance their compositional capability (Lu and Sun, 2013). Therefore, based on the Upper Echelons Theory and the Composition-based View, this study argues that for resource-constrained SRDI SMEs, the innovative integration of internal and external common resources through the construction of compositional capability is an important pathway to improving their innovation performance. Thus, this study introduces compositional capability to explore its mediating role in the relationship between executive cognition and innovation performance in SRDI SMEs in China.

Furthermore, the Dynamic Capability Theory posits that the timely updating of organizational routines can provide a conducive internal environment for the formation and enhancement of enterprises’ dynamic capabilities (Liu et al., 2024; Salvato and Rerup, 2018). Compositional capability is a special dynamic capability particularly suitable for SMEs with limited resources and capabilities (Lu and Sun, 2013). Limited by changes in the execution environment of organizational routines, organizations primarily rely on “variation,” “selection,” and “retention” to achieve routine updates, thereby adapting to new environments, enhancing organizational efficiency, and enabling senior managers’ executive cognition to play a better role in improving the enterprise’s compositional capability (Liu and Yu, 2019). Therefore, this study attempts to explore the moderating effect of organizational routine updating on the relationship between senior managers’ executive cognition and the compositional capability of SRDI SMEs.

In summary, this study makes the following contributions: Theoretically, based on the Composition-based View and Upper Echelons Theory, this paper discusses the influence path of executive cognition on the innovation performance of SRDI SMEs by improving compositional capability. Based on Dynamic Capability Theory, this paper also attempts to study the boundary conditions of executive cognition on innovation performance by analyzing the process of executive cognition enhancing compositional capability under the condition of timely updating of organizational routines, thereby promoting the innovation performance of SRDI SMEs. In practice, this paper provides a development direction for SRDI SMEs to overcome their dilemmas and offers practical guidance and management insights for SRDI SMEs to improve innovation performance.

## 2 Theory and hypothesis

### 2.1 Executive cognition and innovation performance

Executive cognition refers to the process by which senior managers recognize, process, and respond to external environmental information through their cognitive activities (Mitchell et al., 2000; Zhou and Ding, 2024). Uncertainty and instability have become the norm for SRDI SMEs. Executive cognition can guide senior managers to steer the development direction of enterprises in a dynamic environment. Existing research indicates that executive cognition comprises three structural dimensions: willingness cognition, configuration cognition, and ability cognition (Mitchell et al., 2000). Willingness cognition of senior managers in SRDI SMEs refers to their commitment to the SRDI development of the enterprise and the knowledge structure that ensures the realization of this commitment (Yang and Yu, 2016). There are three main types of willingness cognition: innovation opportunity capture, long-term development commitment, and innovation opportunity weighing (Krueger and Brazeal, 1994; Sexton and Bowman-Upton, 1985). Configuration cognition of senior managers in SRDI SMEs refers to the unique individual knowledge structure that supports the SRDI development of enterprises, which can be categorized into four types: patent idea protection, access to external resources, digital network construction, and exclusive skills that are difficult to imitate (Busenitz and Barney, 1997; Macmillan, 1983; Mitchell et al., 2002). Ability cognition of senior managers in SRDI SMEs refers to the knowledge structure that enables them to mobilize skills, knowledge, values, and attitudes to support the SRDI development of enterprises. There are three main types of ability cognition: diagnostic ability, situational knowledge that is not easily imitated, and competence-opportunity matching (Krueger and Carsrud, 1993; Stuart and Abetti, 1990).

Innovation is the core driver of high-quality development for SRDI SMEs (Yang and Xu, 2024; Dong and Li, 2019). In recent years, some scholars have shifted from traditional research on the content and form of innovation to a focus on the returns or outcomes of innovation, thereby strengthening the study of innovation performance. Existing research indicates that a firm’s innovation strategy, innovation resources and capabilities, and innovation knowledge and skills are the key factors affecting innovation performance (Liu et al., 2024).

According to the Upper Echelons Theory, the level of executive cognition affects corporate performance by influencing senior managers’ decisions and behaviors (Hambrick and Mason, 1984; Yang and Xu, 2024). Executive cognition acts as a “lifesaver” for SRDI SMEs in crisis situations. This is because if senior managers possess the ability to quickly identify and absorb effective information and respond keenly, they can make correct decisions swiftly, helping enterprises recover and rebound from crises by improving innovation performance (Zhang et al., 2021; Shan et al., 2021). Specifically: Firstly, the willingness cognition level of senior managers in SRDI SMEs affects the innovation strategy of enterprises. The volatility of the market environment has become the norm, and the high ability to capture innovation opportunities enables senior managers of SRDI SMEs to identify changes in market segment demands more acutely, explore innovation opportunities to meet these needs, take the lead in generating innovation strategies, improve products or services, and make products more specialized and distinctive. As a result, they can serve specific customer groups more precisely (Helfat and Peteraf, 2015). Through opportunity weighing, senior managers can more easily predict potential problems in the implementation of innovation activities in crisis situations, thereby improving the entrepreneurial strategy, enhancing professional technical capabilities and market segmentation abilities more effectively, and avoiding risks in the implementation of innovation activities. The long-term development commitment of senior managers ensures the long-term implementation of the enterprise’s SRDI development strategy, thereby promoting the stable improvement of innovation performance.

Secondly, the innovation and development of SRDI SMEs are more likely to be limited by their own resources and capabilities (Yang and Xu, 2024; Dong and Li, 2019). However, the configuration cognition of senior managers provides resource and capability guarantees for improving enterprise innovation performance. The high protection of patent ideas and exclusive skills by senior managers enables them to distinguish enterprises from competitors by creating novel and distinctive products and services that are difficult to imitate, thereby meeting the needs of customers in market segments and enhancing market segmentation capabilities (Dong and Li, 2019; Shan et al., 2021). The configuration cognition of senior managers helps enterprises obtain the required external resources through resource acquisition and provides subsequent resource acquisition channels for enterprise innovation through network construction, thereby compensating for the disadvantages SRDI SMEs face in obtaining external resources due to scale discrimination, optimizing resource allocation, and accelerating enterprise innovation output (Ma et al., 2022).

Lastly, the high ability cognition of senior managers in SRDI SMEs can provide relevant knowledge and skills to promote professional capabilities. The unique situational knowledge of senior managers can help enterprises diagnose promising market segments, provide knowledge reserves and experience references for market segment development, enhance market segmentation capabilities, and accelerate the speed, frequency, and level of enterprise innovation (Zahra and George, 2002). Innovation is often accompanied by significant uncertainty. The diagnostic ability of senior managers can help enterprises screen out innovation plans that are unsuitable for their professional development, increasing the success probability of feasible plans and enabling the innovation activities of SRDI SMEs to achieve twice the result with half the effort. The competence-opportunity matching function in ability cognition can recombine enterprise personnel, raw materials, or products to make resource allocation more orderly and efficient, thereby producing professional, sophisticated, and unique new products and enhancing professional technical capabilities and system operation capabilities (Guo et al., 2024; Xie and Liu, 2015).

In summary, the willingness cognition, configuration cognition, and ability cognition of executive cognition provide innovation strategies, resources and capabilities, as well as innovation-related knowledge and skills for enterprise innovation, thereby improving the innovation performance of SRDI SMEs. Accordingly, this paper proposes the following hypothesis:

*Hypothesis 1:* Executive cognition has a significantly positive impact on the innovation performance of SRDI SMEs.

### 2.2 Mediating role of compositional capability

Lu and Sun (2013) defined compositional capability as the unique ability of an enterprise to integrate existing internal and external tangible or intangible resources. Under the framework of compositional capabilities, the object of resource integration for SRDI SMEs is primarily common resources rather than heterogeneous resources, and the resource integration process can be divided into two stages: resource acquisition and resource allocation. Resource acquisition is an externally oriented behavior of enterprises, including the identification and acquisition of resources, while resource allocation is an internal behavior of enterprises involving the combination and utilization of resources (Ma et al., 2011).

The Upper Echelons Theory posits that a higher cognitive level of senior managers will help enterprises achieve better resource integration (Hambrick and Mason, 1984). The operating environment of SRDI SMEs is highly uncertain, but such an uncertain environment often conceals opportunities (Yang and Xu, 2024; Liu et al., 2019). A high level of willingness cognition among senior managers can help enterprises capture and weigh opportunities in a timely manner. For SRDI SMEs, it is often difficult to win opportunities relying solely on internal resources. A high level of willingness cognition can help senior managers identify the external resources needed. Commitment limits encourage senior managers to obtain the external resources required for SRDI development. The strong configuration cognition of senior managers can provide a channel for the acquisition of external resources by SRDI SMEs. On one hand, senior managers can use the relationship networks they have built to obtain resources; on the other hand, idea protection and exclusive skills can increase the discourse power and competitiveness of SRDI SMEs in key core areas, thereby expanding their existing relationship networks and increasing access to external resources. When senior managers possess a high level of ability cognition, it means they have rich situational knowledge and diagnostic ability, which helps them allocate resources more reasonably, apply the new resource combination to the business that aligns with their actual market segments through the matching of opportunity and ability, focus on the SRDI development of the main business, and improve the utilization efficiency of enterprise resources. Accordingly, this paper proposes the following hypothesis:

*Hypothesis 2:* Executive cognition has a significantly positive impact on compositional capability of SRDI SMEs.

The lack of resources is the bottleneck of innovation and development for SRDI SMEs (Lian, 2024; Chen et al., 2023). The entire process of producing specialized, refined, and unique products or services requires the support of core resources such as core technology and high-tech talents. The unpredictability of the external environment and the risk of internal production chain disruptions increase the cost and difficulty of cultivating and acquiring core resources, necessitating an effective response through resource integration (Lian, 2024). According to the Composition-based View, for SRDI SMEs that lack heterogeneous resources, they can achieve improved innovation performance through the innovative integration of internal and external common resources (Jian and Kuang, 2020). Specifically, innovation activities require a substantial amount of resource input, which is difficult for SRDI SMEs to meet due to limited internal resources (Chen et al., 2018). SRDI SMEs with compositional capabilities provide resource guarantees for enterprise innovation through the identification and acquisition of external resources. The ability of an SRDI SME to allocate internal and external resources cannot be copied or imitated by its competitors and is also the driving force for the enterprise to improve its performance (Barney, 1991). The innovative and rational allocation of common resources can yield extraordinary benefits and efficiency (Ma et al., 2011). The process of enterprises utilizing resources is also a process in which the value of resources is realized and transformed into enterprise innovation performance (Hitt et al., 2001). Therefore, for SRDI SMEs, their compositional capabilities can significantly enhance their innovation performance. Accordingly, this paper proposes the following hypothesis:

*Hypothesis 3:* Compositional capability has a significantly positive impact on innovation performance of SRDI SMEs.

In summary, based on the Composition-based View, SRDI SMEs can enhance their innovation performance by building compositional capabilities, that is, through the innovative integration of internal and external resources. At the same time, the Upper Echelons Theory suggests that higher cognitive levels among senior managers will help enterprises achieve better resource integration. Therefore, for SRDI SMEs that increasingly struggle to cultivate and acquire core resources, senior managers with high cognitive levels can build compositional capabilities through collaborative integration of internal and external resources, serve specific market segments, improve enterprise operational efficiency, and achieve better innovation performance. Accordingly, this paper proposes the following hypothesis:

*Hypothesis 4:* Compositional capability plays a mediating role between executive cognition and innovation performance of SRDI SMEs.

### 2.3 Moderating effect of organizational routine updating

Organizational routine updating refers to the process in which organizational routines actively “search” when the execution environment of organizational routines changes, thereby adapting organizational routines to the new environment and enhancing the effectiveness of organizational routines (Wang et al., 2012; Salvato and Rerup, 2018). Wang et al. (2012) divided the organizational routine updating into two mechanisms: updating and innovation. The updating mechanism emphasizes the adaptation of internal routines to the environment, which is an optimization process of self-sublation through inheritance and correction. The innovation mechanism refers to the formation of new organizational routines through trial and error in the process of search and selection.

According to the Dynamic Capability Theory, the timely updating of organizational routines can provide a conducive internal environment for the formation and improvement of enterprises’ dynamic capabilities (Salvato and Rerup, 2018). Compositional capability is a special dynamic capability that is particularly suitable for SRDI SMEs that lack resources (Lu and Sun, 2014). In SRDI SMEs, changes in the external environment significantly interfere with and damage the internal rules and regulations of the organization. To adapt to these changes, organizations need to update routines to buffer the negative impact of crises (Liu et al., 2024) and promote the role of senior managers’ cognition in improving the compositional capability of SRDI SMEs (Lu and Sun, 2013; Parmigiani and Jennifer, 2011). Specifically, when the willingness cognition of senior managers acts on the compositional capability of enterprises, organizational routine updating can help enterprises cope with the changing external environment. At this time, the increased awareness of crisis among senior managers will prompt them to question and reflect on the issues in enterprise development, thereby promoting their willingness cognition to recognize and obtain the resources lacking in enterprise development. This lays a resource foundation for the construction of compositional capability in SRDI SMEs. When the configuration cognition of senior managers acts on the compositional capability of SRDI SMEs, organizational routines can provide a conducive organizational environment for senior managers’ allocation cognition to take effect through the updating and innovation mechanisms, thereby attracting partners and helping enterprises obtain external resources. When the ability cognition of senior managers acts on the compositional capability of enterprises, the innovation mechanism of organizational routine updating enables senior managers to break the routine, accept new ideas, and propose novel solutions to problems. This helps enterprises generate more innovative and valuable resource allocation methods, increase resource utilization efficiency, and thus enhance the compositional capability of SRDI SMEs. In other words, when the level of organizational routine updating is low, organizational routines are stable, and fixed standard consensus and behavior patterns are evident, which can easily lead to myopia among senior managers and rigidity in the organization. As a result, enterprises will struggle to withstand various internal and external shocks (Salvato and Rerup, 2018; Zhou and Wu, 2010). At this time, even high executive cognition among senior managers in SRDI SMEs will find it difficult to lead the enterprises to form the compositional capabilities of innovatively integrating internal and external resources. Accordingly, this paper proposes the following hypothesis:

*Hypothesis 5:* Organizational routine updating positively moderates the relationship between executive cognition and compositional capability in SRDI SMEs. When the level of organizational routine updating is higher, the relationship between executive cognition and compositional capability is stronger; The reverse is weaker.

**Figure 1:**
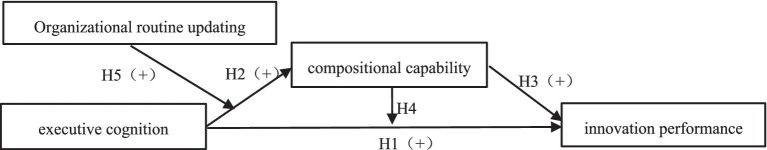
Research model.

The theoretical model of this study is shown in Figure 1.

## 3 Research design

### 3.1 Research object and investigation process

The sample enterprises in this study are SRDI SMEs identified by the provincial departments of Industry and Information Technology in China, primarily focusing on information transmission, software and information technology services, and manufacturing industries. According to statistical data from the Ministry of Industry and Information Technology of China, as of July 2024, there were 140,000 SRDI SMEs nationwide. Shandong, Jiangsu, and Zhejiang—three economically developed provinces in China—collectively accounted for 31.3% of the national total of such enterprises. Considering the social network connections of the research team, 98 SRDI SMEs from these three provinces were selected as research subjects, with 4–5 senior executives from each enterprise chosen as study participants. Starting from May 2023, questionnaires were distributed offline, through WeChat, and via email. In the first stage, variables such as executive cognition, compositional capability, and organizational routine updating were investigated. Three months later, the second stage of the survey was carried out, mainly distributing questionnaires related to the dependent variable, innovation performance. A total of 482 questionnaires were distributed, and 409 valid sample questionnaires from 98 enterprises were finally obtained after eliminating those with obvious errors and incomplete filling, with an effective recovery rate of 84.8%. The analysis results of sample characteristics are shown in Tables 1, 2.

**Table 1:** Overview of the surveyed senior managers.

Variables		Proportion of variables (%)
Gender	Male	54.8
	Female	45.2
Education level	Junior college and below	20.3
	Undergraduate	65.8
	Master and above	13.9

**Table 2:** Overview of the surveyed SRDI SMEs.

Enterprise characteristics	Percentage	Enterprise characteristics	Percentage
	(%)		(%)
Enterprise size (operating revenue in 2023)		Number of employees	
Less than RMB60 million (excluding RMB60 million)	4.9	199 or less	24.9
60–80 million yuan (excluding 80 million yuan)	18.6	200–399 people	37.9
80–100 million yuan (excluding 100 million yuan)	20.1	400–599 people	21.8
100–30,000 yuan (excluding 300 million yuan)	39.6	600–799 people	14.4
More than 300 million yuan	16.8	800 and above	1.0
Time of establishment		R&D investment as a percentage of sales revenue	
Less than 15 years (excluding 15 years)	24.2	3–6% (excluding 6%)	16.3
15–19 years	60.1	6–10% (excluding 10%)	68.4
20–24 years	12.8	10% and above	15.3
25 years and above	2.9		

### 3.2 Variable measurement

Based on the reference of mature scales at home and abroad, the measurement items of this study are adjusted and modified according to expert opinions, the business scenarios of Chinese enterprises and the pre-survey results. Except for the control variables, the five-point Likert scale is used for measurement. The specific variables are described as follows.

The executive cognition, the scale was developed by Smith et al. (2010). First, the researcher translated the scale into Chinese according to the language expression habits of Chinese and solicited expert opinions, and then another researcher translated it into English. After repeated comparison and correction, the final Chinese questionnaire was determined, and then the pre-survey was conducted. According to the pre-survey data processing results, the items with factor loading <0.6 are deleted, and the executive cognition scale with 15 items is finally determined. Specific items include “We have the ability to obtain a variety of capital sources for the development and expansion of the enterprise” and so on. In this study, the Cronbach’s *α* coefficient of the scale is 0.952.

Compositional capability, it refers to the measurement of compositional capability by Lu and Sun (2014) and is modified according to the predictive quantity feedback, including four items. According to the feedback of the respondents, the language expression of some items is not concise and clear. According to the feedback, the item “The enterprise is good at adding externally obtained resources into the ever-changing portfolio of existing resources” was modified to “The enterprise is good at innovatively integrating externally obtained resources with existing resources,” and the item “Your company is good at replacing original resources with cost-effective resources. Improve efficiency and reduce costs” was adjusted to “The enterprise is good at reducing costs and improving profits through the integration of internal and external resources.” In this study, the Cronbach’s α coefficient of the scale is 0.942.

Innovation performance draws on the scale of Zhang and Li (2010) and Yu and Cai (2013). According to the pre-survey data processing results, we deleted the items with a lower factor loading of 0.6, which contains 4 items in total. In this study, the Cronbach’s α coefficient of the scale is 0.930.

Organizational routine updating, it was developed by Wang et al. (2012). According to the pre-survey results, the items with factor loading <0.6 are removed, and five measurement items are finally determined. Specific items include “The enterprise encourages employees to participate in the revision process of organizational norms” and so on. In this study, the Cronbach’s α coefficient of the scale is 0.945.

Control variables, based on existing research on innovation performance, firm size (measured by the number of employees: 1 means “199 or less,” 2 means “200–399,” 3 means “400–599,” 4 means “600–799,” 5 means “800 or more”), company age (1 means “<15 years “, 2 means “16–19 years,” 3 means “20–24 years,” 4 represents “25 years or more”) and the proportion of R&D investment in sales revenue (1 represents “<3–6% (excluding 6%),” 2 represents “6–10% (excluding 10%),” and 3 represents “10% or more”) are set as the control variables of this study.

### 3.3 Statistical analysis

In this study, SPSS 26.0 and MPLUS 7.4 were used for statistical analysis of the data. The specific statistical analysis process is as follows: First, SPSS 26.0 and MPLUS 7.4 were used to conduct confirmatory factor analysis on the four variables involved in the study. Secondly, SPSS 26.0 was used for descriptive statistical analysis and correlation analysis. Finally, hierarchical regression analysis was used to test the hypotheses.

## 4 Research results

### 4.1 Common method bias test

Although this study conducted a questionnaire survey on the research variables in two stages, and filled in the questionnaire anonymously, the common method bias cannot be completely avoided because all the variables of the same sample were filled in by the same respondent. Therefore, this paper adopts two methods to test. First, through confirmatory factor analysis, we test whether most of the differences in the data can be explained by a single factor. The results show that the fitting index is very unsatisfactory: *χ*^2^ = 1637.534, df = 351, *χ*^2^/df = 4.665, RMAEA = 0.119, CFI = 0.688, TLI = 0.662, SRMR = 0.096, and it can be inferred that there is no serious common method bias in this study. Secondly, the correlation coefficient between the variables is tested. Table 3 shows that the correlation coefficient between variables is <0.9, which again indicates that there is no serious common method bias in the sample data.

**Table 3:** Descriptive statistics and correlation analysis.

Variables	1	2	3	4	5	6	7
1. Enterprise age	–						
2. Enterprise scale	0.200^*^	–					
3. Proportion of R&D investment	0.047	−0.057	–				
4. Executive cognition	−0.176	−0.424^**^	0.010	0.915			
5. Compositional capability	−0.201^*^	−0.486^**^	0.035	0.765^**^	0.898		
6. Innovation performance	−0.304^**^	−0.432^**^	0.055	0.789^**^	0.880^**^	0.882	
7. Organizational routine updating	−0.133	−0.511^**^	0.109	0.811^**^	0.814^**^	0.811^**^	0.881
Average value	1.950	2.311	1.658	4.100	3.974	4.091	4.159
Standard deviation	0.404	0.641	0.366	0.332	0.444	0.477	0.425

*represents the significance level of *P* < 0.05, and **represents the significance level of *P* < 0.01, the same below; values in diagonal brackets are the square root of AVE.

### 4.2 Reliability and validity test

In this study, the software SPSS26.0 and MPLUS7.4 are used to conduct confirmatory factor analysis to test the reliability and validity of the research variable scale. In the first step, the reliability of the scale is tested by combining reliability and internal consistency coefficients (CR value and Cronbach’s a coefficient). As shown in Table 4, the CR value and Cronbach’s a coefficient of each variable all exceed 0.8, indicating that the reliability of each variable scale is good.

**Table 4:** Reliability and validity test results of the scales.

Variables	Cronbach’s a coefficient	CR	AVE
Executive cognition	0.952	0.939	0.838
Compositional capability	0.942	0.943	0.806
Innovation performance	0.930	0.875	0.778
Organizational routine updating	0.945	0.946	0.777

The second step is to test the validity of the scale by comparing the AVE value of each variable and the correlation coefficient between the square root of the AVE of each variable and other variables. As can be seen from Table 4, the AVE of each variable is higher than 0.5, indicating that the variables all have good convergent validity. Moreover, as shown in Table 3, the square roots of the AVE (Average Variance Extracted) for all variables are greater than their correlation coefficients with other variables. And compared to other factor models, the four-factor model demonstrates the best fit (*χ*^2^ = 659.833, df = 344, *χ*^2^/df = 1.918, RMSEA =0.062, CFI = 0.918, TLI =0.908, SRMR =0.065), meeting the recommended model fit criteria. These results collectively indicate that the variables exhibit significant discriminant validity.

### 4.3 Aggregate analysis

Since the survey data providers are individual senior managers in enterprises, in order to effectively avoid the deviation of model parameter estimates caused by the interaction between individuals and accurately measure variables, it is necessary to test the intra-group consistency and inter-group heterogeneity of variables. The results are shown in Table 5, ICC1 of each variable is >0.1, ICC2 is >0.5, and Rwg is > 0.7. Therefore, the sample data can be aggregated to the enterprise level, and 98 enterprises are taken as samples, and the average of the survey results of senior managers in each enterprise is used as the observation quantity of each variable.

**Table 5:** Index values of the aggregate analysis of each variable.

Variables	Rwg	ICC_1_	ICC_2_
Executive cognition	0.871	0.218	0.538
Compositional capability	0.901	0.243	0.572
Innovation performance	0.852	0.243	0.573
Organizational routine updating	0.907	0.282	0.621

### 4.4 Descriptive statistical analysis and correlation analysis

The results of the descriptive statistical analysis of this study are shown in Table 3. It can be seen from Table 3 that executive cognition is significantly positively correlated with compositional capability (*r* = 0.765, *p* < 0.01) and innovation performance (*r* = 0.789, *p* < 0.01). At the same time, the compositional capability is also significantly positively correlated with the innovation performance (*r* = 0.880, *p* < 0.01). These results provide a preliminary basis for the hypothesis tests that follow.

### 4.5 Hypothesis test results

This study employs linear regression analysis to test the hypotheses, along with the Bootstrap method for robustness checks. During the regression analysis, multicollinearity issues were simultaneously examined. As shown in Tables 6, 7, the variance inflation factor (VIF) values for all variables are significantly <10, indicating the absence of severe multicollinearity problems.

**Table 6:** Regression results of main effect.

Variables	Compositional capability				Innovation performance					
	M1		M2		M5		M6		M7	
	*β*	VIF	*β*	VIF	*β*	VIF	*β*	VIF	*β*	VIF
Control variables										
Enterprise age	−0.127	1.043	−0.053	1.056	−0.250^*^	1.043	−0.172^**^	1.056	−0.147^**^	1.063
Enterprise scale	−0.379^***^	1.044	−0.119	1.208	−0.307^**^	1.044	−0.032	1.208	−0.001	1.219
Proportion of R&D investment	0.033	1.005	−0.005	1.008	0.14	1.005	0.1	1.008	0.113^*^	1.006
Independent variable										
Executive cognition			0.702^***^	1.195			0.742^***^	1.195		
Compositional capability									0.807^***^	1.217
*R^2^*	0.178		0.590		0.198		0.659		0.734	
adjusted *R^2^*	0.152		0.573		0.173		0.644		0.723	
*ΔR^2^*	0.178		0.412		0.198		0.461		0.075	
*F*	6.791^***^		33.509^***^		7.752^***^		44.882***		64.160***	

***represents the significance level of *P* < 0.001.

**Table 7:** Regression analysis of mediating and moderating effects.

Variables	Compositional capability				Innovation performance	
	M3		M4		M8	
	*β*	VIF	*β*	VIF	*β*	VIF
Control variables						
Enterprise age	−0.073	1.06	−0.054	1.08	−0.142**	1.063
Enterprise scale	−0.033	1.282	−0.01	1.311	0.034	1.243
Proportion of R&D investment	−0.006	1.008	0.008	1.019	0.103*	1.008
Independent variable						
Executive cognition	0.247*	3.299	0.329**	3.657	0.351***	2.398
Compositional capability					0.556***	2.441
Moderator variable						
Organizational routine updating	0.583***	3.451	0.543***	3.537		
Executive cognition × Organizational routine updating			0.148*	1.173		
*R^2^*	0.689		0.707		0.785	
adjusted *R^2^*	0.672		0.688		0.774	
*ΔR^2^*	0.129		0.018		0.051	
*F*	40.728***		36.679***		67.378***	

*represents the significance level of *p* < 0.05, **represents the significance level of *p* < 0.01, ***represents the significance level of *p* < 0.001.

#### 4.5.1 Main effect test

This study primarily tests Hypothesis 1, Hypothesis 2, and Hypothesis 3 using hierarchical regression analysis conducted with SPSS 26.0: (1) The control variables (enterprise age, enterprise scale, proportion of R&D investment) and independent variables (executive cognition) are introduced into the regression equation successively to analyze the impact of executive cognition on innovation performance. (2) Control variables and independent variables (executive cognition) are introduced in turn to analyze the impact of executive cognition on compositional capability. (3) Control variables and mediating variables (compositional capability) are introduced in turn to analyze the impact of compositional capability on innovation performance. The results are shown in Table 6. According to Model 6, executive cognition can significantly promote the improvement of enterprise innovation performance (*β* = 0.742, *p* < 0.001), so Hypothesis 1 is verified. In Model 2, executive cognition has a significant effect on improving the compositional capability (*β* = 0.702, *p* < 0.001), so Hypothesis 2 is verified. Model 7 shows that the compositional capability has a significant positive impact on innovation performance (*β* = 0.807, *p* < 0.001), so Hypothesis 3 is verified.

In addition, Bootstrap testing was conducted using Mplus 7.4 with 5,000 random samples. The impact coefficient of executive cognition on innovation performance is 0.272, with a 95% confidence interval of [0.102, 0.443], which does not include zero, indicating a significant direct effect. The impact coefficient of executive cognition on compositional capability is 0.522, with a 95% confidence interval of [0.282, 0.762], which does not include zero, indicating a significant direct effect. The impact coefficient of compositional capability on innovation performance is 0.481, with a 95% confidence interval of [0.297, 0.664], which does not include zero, indicating a significant direct effect. Therefore, Hypothesis 1, Hypothesis 2, and Hypothesis 3 are further supported.

#### 4.5.2 Mediating effect test

This study primarily tests Hypothesis 4 using hierarchical regression analysis conducted with SPSS 26.0. Control variables (enterprise age, enterprise scale, proportion of R&D investment), the independent variable (executive cognition), and the mediating variable (compositional capability) were sequentially introduced into the regression equation. The results are shown in Table 7. By comparing Model 6 and Model 8, it is evident that after including both executive cognition and compositional capability in the regression equation, compositional capability still significantly and positively influences innovation performance (Model 8, *β* = 0.556, *p* < 0.001), and executive cognition continues to significantly affect innovation performance (Model 8, *β* = 0.351, *p* < 0.001). However, the regression coefficient for executive cognition significantly decreases, indicating that compositional capability partially mediates the relationship between executive cognition and innovation performance. Thus, Hypothesis 4 is preliminarily supported.

Additionally, this study employs Bootstrap testing in Mplus 7.4 to examine the mediating effect of compositional capability between executive cognition and firm innovation performance. The number of random samples was set to 5,000, and the bias-corrected method was used to estimate the upper and lower bounds of the effect interval, with a 95% confidence interval. If the confidence interval does not include zero, the mediating effect is considered significant. With innovation performance as the dependent variable, executive cognition as the independent variable, and compositional capability as the mediating variable, the indirect effect is 0.251, with a Bootstrap confidence interval of [0.126, 0.372], which does not include zero. Therefore, Hypothesis 4 is further validated.

#### 4.5.3 Moderating effect test

In order to verify the interaction between executive cognition and organizational routine updating, on the basis of setting compositional capability as the dependent variable, the control variable, independent variable (executive cognition) and moderating variable (organizational routine updating) are introduced into the regression equation, and finally the standardized interaction term between independent variable and moderating variable is put in. The results are shown in Table 7. According to Model 4, the interaction between executive cognition and organizational routine updating has a significant positive impact on compositional capability (*β* = 0.148, *p* < 0.05), which indicates that organizational routine updating can positively regulate the relationship between executive cognition and compositional capability. When the level of organizational routine updating is high, the positive impact of executive cognition on compositional capability is greater. When the level of organizational routine updating decreases, the positive impact of executive cognition on compositional capability will be weakened. Therefore, Hypothesis 5 is verified.

**Figure 2:**
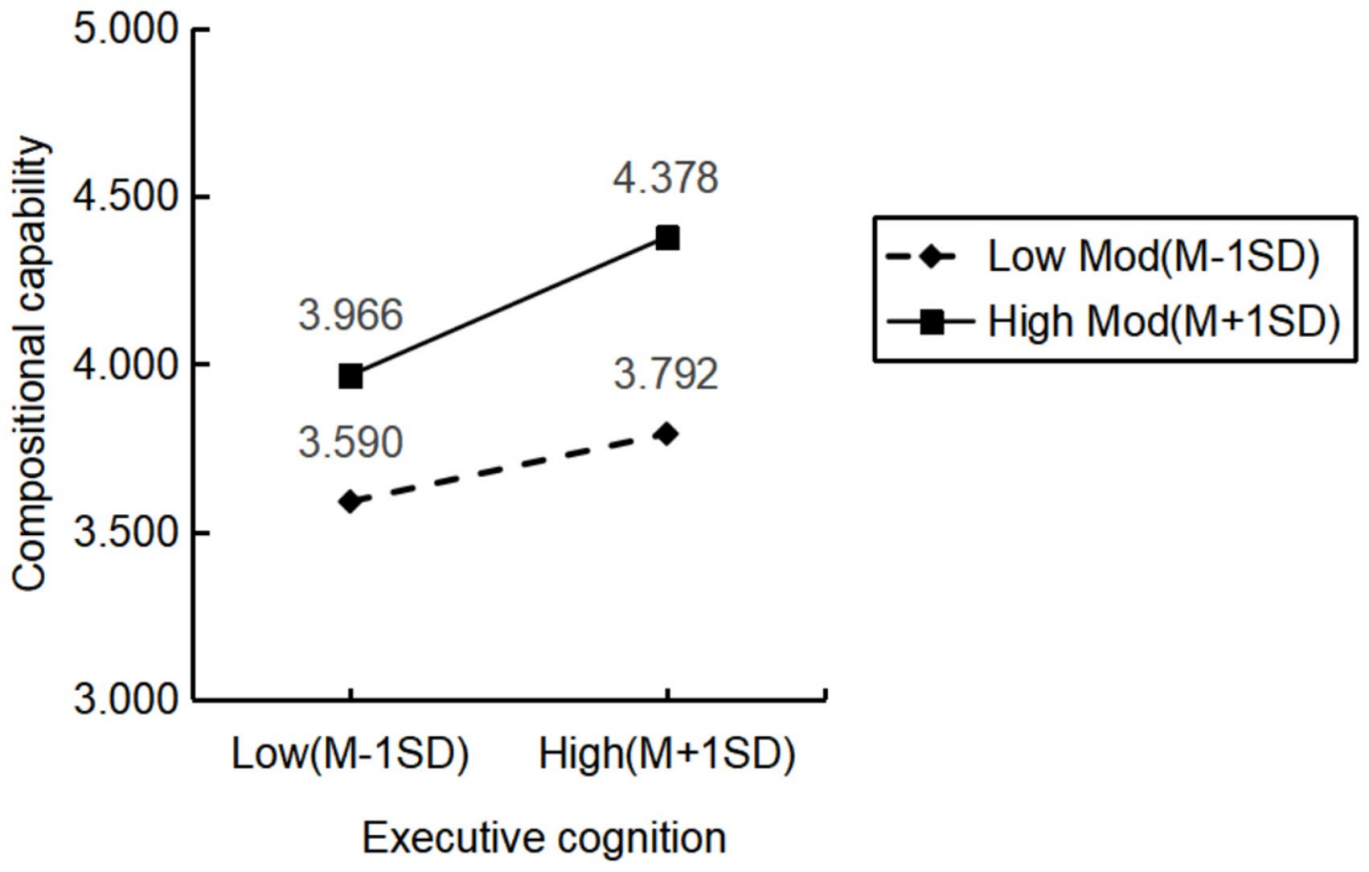
Moderating effect diagram.

Furthermore, Mplus 7.4 was used to further validate the moderating effect of organizational routine updating. Organizational routine updating was divided into a high-score group (mean + standard deviation) and a low-score group (mean - standard deviation) to examine the differences in the moderating effects between the two groups. In the high-score group of organizational routine updating, the impact coefficient of executive cognition on compositional capability is 0.622, with a 95% confidence interval of [0.487, 0.758], which does not include zero, indicating a significant positive effect. In the low-score group of organizational routine updating, the impact coefficient of executive cognition on compositional capability is 0.306, which, although still significant, is much smaller than that of the high-score group. More importantly, the between-group difference coefficient is 0.316, with a 95% confidence interval of [0.164, 0.468], which does not include zero, indicating a significant difference. Therefore, Hypothesis H5 is further supported. At the same time, in order to more intuitively show the interaction effect, one standard deviation higher than and lower than the mean value is used as the benchmark to describe the influence of executive cognition on compositional capability under different levels of organizational routine updating. As shown in Figure 2, the positive impact of the interaction effect at high levels of organizational routine updating is evident.

## 5 Discussion

Based on Upper Echelon Theory and Composition-based View, this paper constructs the research framework of executive cognition on innovation performance in SRDI SMEs, and draws the following conclusions: (1) Executive cognition has a significant role in improving enterprise innovation performance. (2) Compositional capability plays a mediating role in the relationship between executive cognition and enterprise innovation performance. (3) The organizational routines updating positively moderates the effect of executive cognition on compositional capability. That is, when the level of organizational routine updating is high, the positive effect of executive cognition on compositional capability is strengthened. On the contrary, the positive effect of executive cognition on compositional capability is weakened.

### 5.1 Theoretical significance

1. The research reveals the black box of the influence mechanism of executive cognition on the innovation performance of SRDI SMEs, and enriches the relevant theoretical research of SRDI SMEs. Previous literature has shown that executive cognition has an important impact on innovation performance, but few studies have explored whether the previous research conclusions are applicable to SRDI SMEs and the influence mechanism. This study confirms that SRDI SMEs urgently need senior managers with high cognitive level to guide the development direction of the enterprise in the dynamic environment and improve the innovation performance of the enterprise. In this process, SRDI SMEs have limited resources and capabilities, It is necessary for senior managers to build compositional capability through their executive cognition to creatively integrate internal and external resources to achieve better innovation performance.
2. The research verifies the important value of the compositional capability in the process of improving the innovation performance of SRDI SMEs, and further enriches and improves the Composition-based View. It has been argued that Composition-based View can guide enterprises that lack core resources to fully integrate and coordinate internal and external common resources by building compositional capability, so as to improve their innovation performance. However, it has not yet answered what scale and type of enterprises the Composition-based View is specifically applicable to, and how senior managers can help enterprises build and improve the compositional capability. This paper finds that due to the lack of resources and the characteristics of SRDI SMEs, their development is easily constrained by the “bottleneck” technology, and they need to innovate and integrate common resources by building compositional capabilities. Therefore, the Composition-based View is especially suitable for guiding the innovation development of SRDI SMEs.
3. The research significantly broadens the application scope and boundary conditions of the Upper Echelons Theory. By applying this theory to the context of innovation performance in SRDI SMEs, we explore how executive cognition influences innovation performance through compositional capability. Moreover, we investigate the interaction between organizational routines updating and executive cognition on compositional capability. Our findings reveal that timely updates to organizational routines can significantly amplify the positive impact of executive cognition on compositional capability. In this way, this study not only extends the theoretical applicability of the Upper Echelons Theory but also clarifies its boundary conditions in the context of SRDI SMEs.

### 5.2 Management implications

1. Enhancing Executive Cognition in SRDI SMEs. The current complex external environment presents significant challenges to the SRDI SMEs. However, crises often coexist with opportunities, revealing innovative prospects in new business models, operational paradigms, and emerging technologies. In this context, senior executives of SRDI SMEs must establish an innovation-centric cognitive system. By engaging in continuous learning, accumulating practical experience, and enriching their knowledge base, they can develop and refine a structured knowledge framework. This will enhance their cognitive capabilities, enabling them to translate strategic insights into actionable innovation roadmaps in a timely manner. As a result, enterprises can seize emerging opportunities, persist in innovation-driven activities, and prioritize the improvement of innovation performance.
2. Emphasize the Development of Compositional Capability. Senior managers of SRDI SMEs should prioritize cultivating compositional capability as a key driver for enterprise growth. To achieve this, they must leverage social networks and continuously break through organizational boundaries to identify and acquire essential resources for development. Additionally, they should enhance the ability to creatively combine both internal and external resources. Firstly, a resource coordination network can be established to strengthen industry-university-research cooperation. By tapping into regional industrial clusters, enterprises can access shared technical service platforms, thereby reducing the costs associated with resource integration. Secondly, optimizing the organizational mechanism is crucial. A flexible management structure should be built, and cross-departmental project teams should be implemented to improve the efficiency of resource allocation. Thirdly, digital transformation can be harnessed to connect with industrial Internet platforms. This will enable the integration of production and supply chain data, facilitate equipment networking and operational visualization, and transform ordinary data into valuable resources for management decision-making.
3. Conduct Timely Organizational Routine Updating. SRDI SMEs should proactively update their organizational routines to keep pace with the ever-changing business environment. This is essential for ensuring that executive cognition can effectively drive the development of the enterprise’s compositional capabilities. To achieve this, enterprises must adopt a dual focus: externally, they should prioritize the “search” and “selection” of advanced external routines, actively identifying and integrating best practices from the broader industry. Internally, they should encourage improvisational behaviors among organizational members, fostering a culture of agility and innovation. By discarding outdated elements of old routines while retaining their core strengths, enterprises can enhance the flexibility and adaptability of their organizational processes. This dual approach not only maximizes the impact of executive cognition but also strengthens the enterprise’s ability to absorb and integrate resources, ultimately contributing to sustainable growth and competitive advantage.

### 5.3 Deficiencies and prospects

Despite its contributions, this study has several limitations that warrant consideration. First, the SRDI SMEs examined in this study primarily belong to industries such as information transmission, software and information technology services, and manufacturing, and are limited to enterprises in East China. This narrow focus means that the influence of industry-specific factors and regional cultural differences cannot be fully excluded. Future research could extend the scope to other industries and regions to enhance the generalizability of the findings. Second, this study relies on cross-sectional data to empirically analyze how executive cognition influences innovation performance in SRDI SMEs. While valuable, cross-sectional data inherently limit our ability to capture dynamic relationships and establish robust causal inferences. Future research could employ grounded theory approaches, case study methodologies, or longitudinal dynamic data to conduct time-series analyses of SRDI SMEs. Such methods would allow for a deeper and more comprehensive exploration of the relationship between executive cognition and innovation performance, ultimately providing more actionable recommendations for enhancing innovation outcomes in these enterprises. Third, this study focuses solely on executive cognition, a senior manager trait, and does not account for the potential impact of other characteristics of top managers on innovation performance. Future research could incorporate additional top-manager traits as control variables in the research model, thereby distinguish the unique role of executive cognition in driving innovation performance in SRDI SMEs.

## Data Availability

The original contributions presented in the study are included in the article/supplementary material, further inquiries can be directed to the corresponding authors.
